# Crystal structure and supra­molecular features of bis­{ethyl 2-[1-methyl-3-(pyridin-2-yl)-1*H*-1,2,4-triazol-5-yl]acetate}­tri­nitratolanthanum(III)

**DOI:** 10.1107/S2056989025005419

**Published:** 2025-06-24

**Authors:** Valeriia Halushchenko, Oleksandr Korovin, Natalya Rusakova, Viktoriya Dyakonenko, Dmytro Khomenko, Rostyslav Lampeka, Serhii Smola

**Affiliations:** a A. V. Bogatsky Physico-Chemical Institute of the National Academy of Sciences of Ukraine 86 Lyustdorfska doroga Odessa Ukraine; b SSI "Institute for Single Crystals", National Academy of Sciences of Ukraine, Nauky ave. 60, 61001 Kharkiv, Ukraine; c V. I. Vernadskii Institute of General and Inorganic Chemistry, National Academy of Sciences of Ukraine, Akad. Palladin Ave 32/34, Kyiv 03142, Ukraine; d Department of Chemistry, Taras Shevchenko National University of Kyiv, Volodymyrska str. 64/13, 01601 Kyiv, Ukraine; e Enamine Ltd. (www.enamine.net), Winston Churchill str. 78, 02094 Kyiv, Ukraine; Universidad de la República, Uruguay

**Keywords:** crystal structure, lanthanum(III) complex, pyridinyl-1,2,4-triazole, inter­molecular inter­actions

## Abstract

The title lanthanum(III) complex, [La(Et-MPTA)_2_(NO_3_)_3_] {where Et-MPTA is ethyl 2-[1-methyl-3-(pyridin-2-yl)-1*H*-1,2,4-triazol-5-yl]acetate} or [La(C_24_H_28_N_8_O_4_)(NO_3_)_3_], crystallizes in the monoclinic space group *C*2/*c* (No. 15). The lanthanum atom is twelve-coordinate, bonded to two oxygen atoms from carboxyl­ate groups, four nitro­gen atoms from two pyridinyl-1,2,4-triazole ligands, and six oxygen atoms of three NO_3_^−^ anions. The coordination geometry around the lanthanum atom can be described as a distorted icosa­hedron. Supra­molecular features include π-stacking inter­actions between pyridyl and triazole rings and weak inter­molecular N—O⋯C inter­actions, which lead to the formation of layers parallel to the (101) plane.

## Abstract 2

The crystal structure of a mononuclear 12-coordinate lanthanum(III) complex with a pyridinyl-1,2,4-triazole derivative is reported and discussed.

## 1. Chemical context

Triazole-based compounds have wide applications in various fields such as medicine, materials science, and pharmaceuticals (Morais *et al.*, 2022 ▸). The variation of the substituents on the triazole ring allows the creation of a broad range of functional materials. Ligands containing the 1,2,4-triazole fragment coordinate through nitro­gen donor centers, and complexes with 1,2,4-triazole ligands may exhibit photoluminescence (Matin *et al.*, 2022 ▸; Schweinfurth *et al.*, 2017 ▸). Rare-earth metal complexes with nitro­gen-containing ligands have garnered significant inter­est due to their potential applications in various fields including catalysis, luminescence, and magnetic materials (Kainat *et al.*, 2024 ▸; Kaczmarek *et al.*, 2018 ▸; Zeybel & Köse, 2023 ▸).
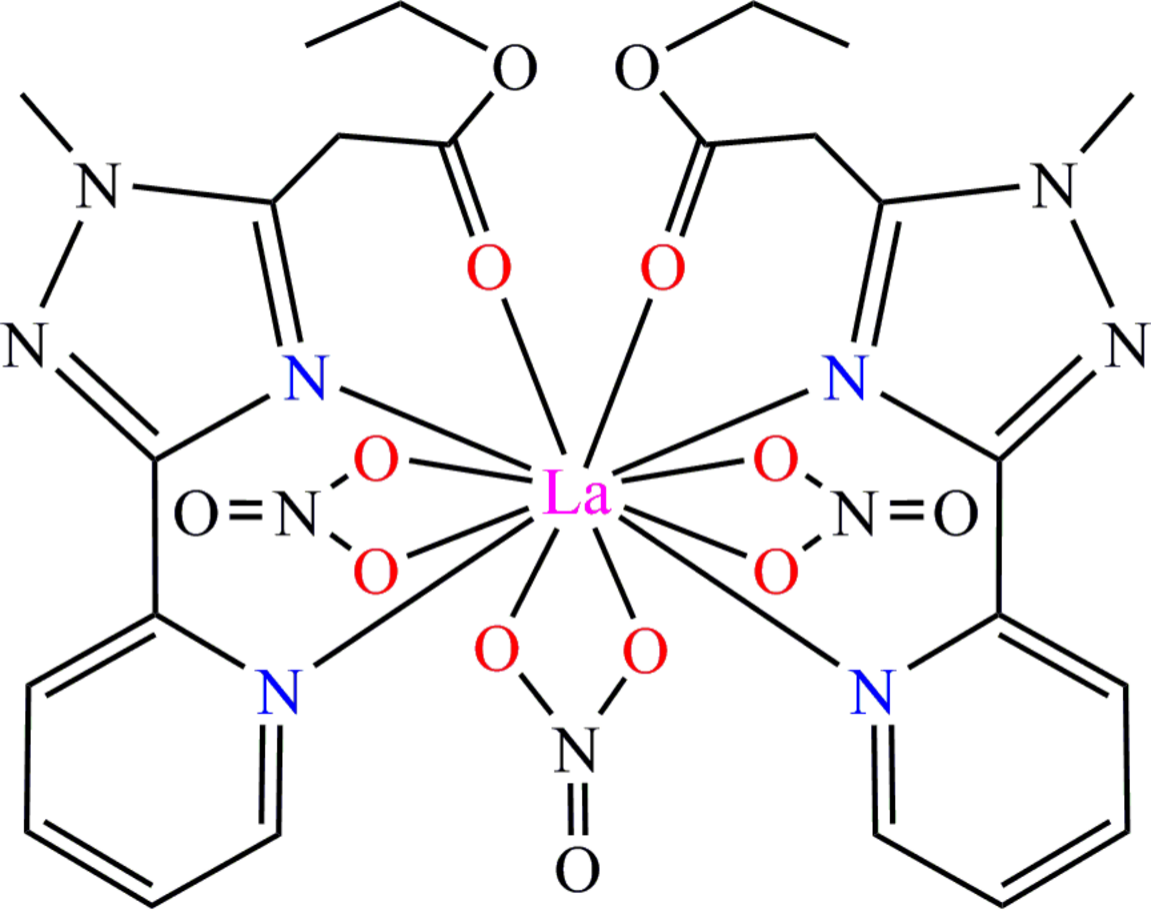


The pyridinyl-1,2,4-triazole derivative used in this study, namely ethyl 2-[1-methyl-3-(pyridin-2-yl)-1*H*-1,2,4-triazol-5-yl]acetate, is a versatile ligand that can coordinate to metal ions in different modes, leading to diverse structural motifs. The investigation of the crystal structure of the lanthanum(III) complex with this ligand provides insights into lanthanide coordination chemistry and the supra­molecular inter­actions that consolidate the crystal structure. Understanding these structural features is crucial for designing new lanthanide-based materials with tailored properties.

## 2. Structural commentary

**Figure 1:**
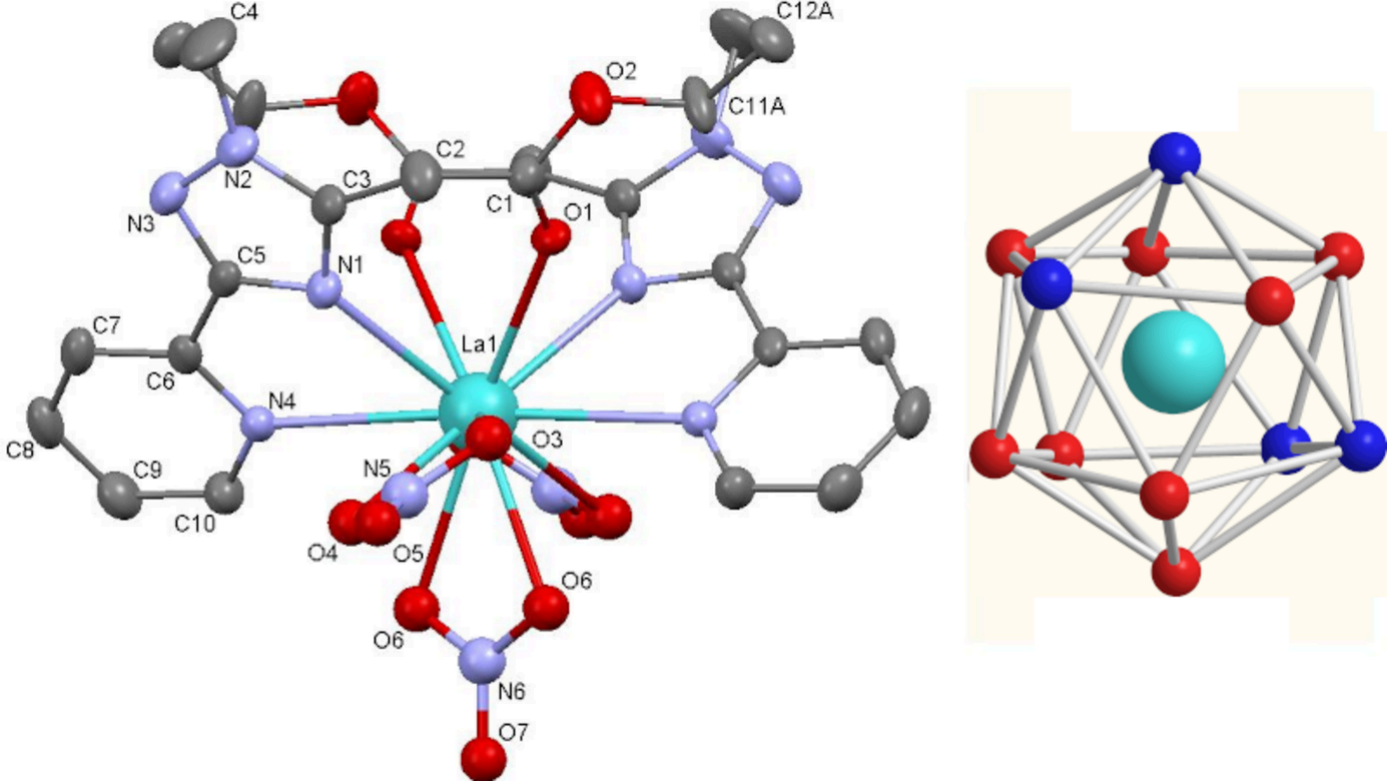
Mol­ecular structure of the title compound. Hydrogen atoms and the disordered C11*B*, C12*B* atoms are omitted for clarity. Inset: icosa­hedral coordination environment around the La^III^ atom.

The title [La(Et-MPTA)_2_(NO_3_)_3_] complex crystallizes in the monoclinic space group *C*2/*c* with the complex occupying a special position, at which the central lanthanum atom is located on a twofold axis. The coordination geometry around the lanthanum atom can be described as a distorted icosa­hedron (Fig. 1 ▸ inset), which is common for 12-coordinated *Ln*^III^ complexes (Jing *et al.*, 1994 ▸; Jones *et al.*, 1997 ▸; Chandrasekhar *et al.*, 2009 ▸). The coordination polyhedron is formed by two oxygen atoms from the carboxyl­ate groups, four nitro­gen atoms from two pyridinyl-1,2,4-triazole of Et-MPTA ligands, and six oxygen atoms of three coordinated NO_3_ anions (Fig. 1 ▸). The La—O bond lengths range from 2.648 (2) to 2.752 (2) Å, and the La—N bond lengths range from 2.735 (2) to 2.841 (2) Å (Table 1 ▸). These bond lengths are consistent with those reported for other lanthanum(III) complexes with nitro­gen- and oxygen-donor ligands (Guillaumont, 2006 ▸; Mishra, 2008 ▸; Cotton *et al.*, 2022 ▸). Three NO_3_^−^ anions are coordinated to La^III^ ion via oxygen atoms in a terminal bidentate manner (de Bettencourt-Dias *et al.*, 2012 ▸). One nitrate group is coordinated in a symmetric manner where the La—O6 bond length and its symmetry equivalent are both 2.648 (2) Å. Two other nitrate groups have asymmetric type of coordination with the La—O3 and La—O4 bond lengths equal to 2.658 (3) Å and 2.753 (3) Å, respectively.

## 3. Supra­molecular features

**Figure 2:**
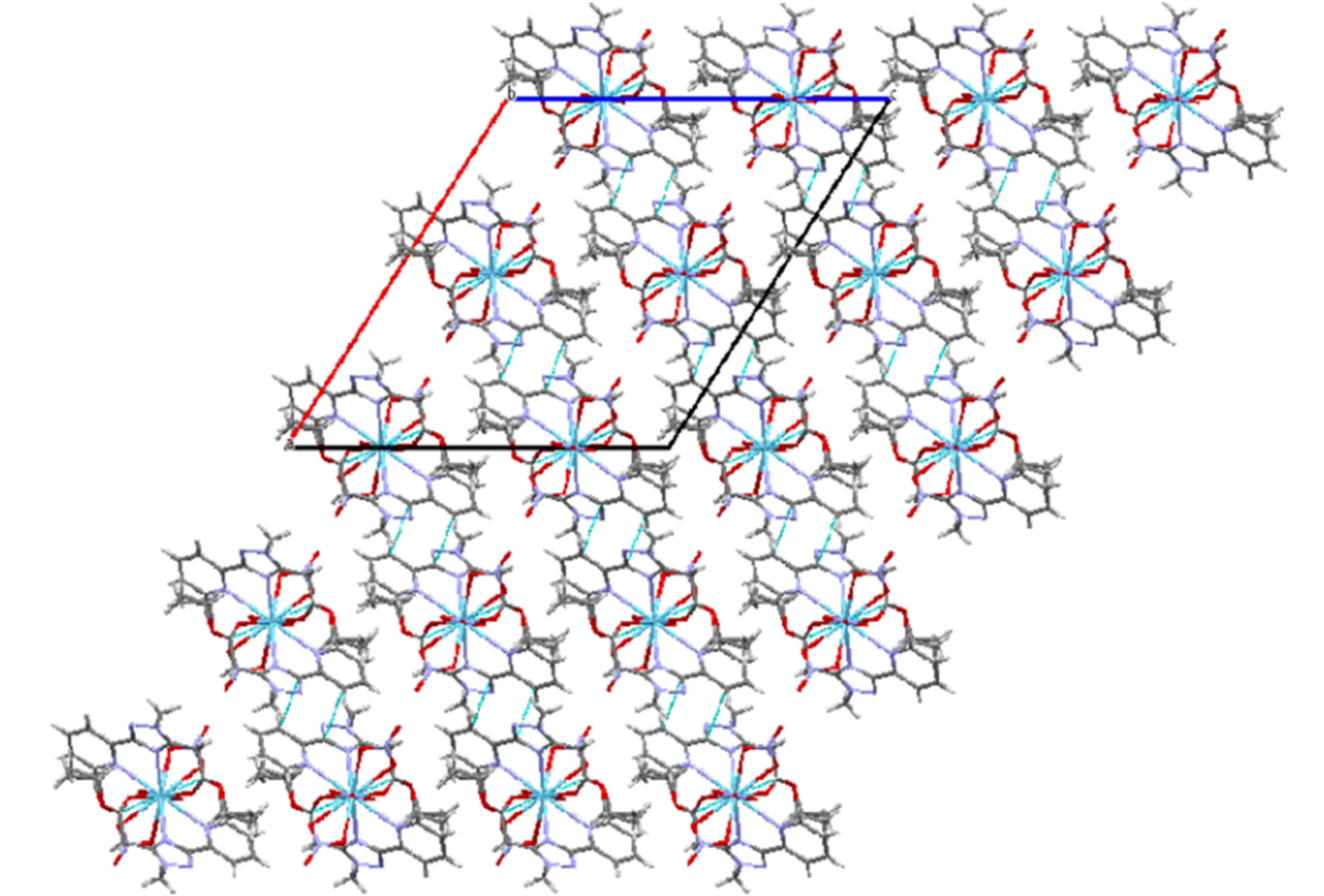
Crystal packing view of [La(Et-MPTA)_2_(NO_3_)_3_] along the *b* axis.

In the crystal, π-stacking inter­actions are observed between the pyridyl substituent and the triazole ring [C5⋯C7 = 3.283 (5) Å, *Cg*1⋯*Cg*2(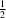 − *x*, 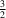 − *y*, 1 − *z*) = 3.809 (2) Å where *Cg*1 and *Cg*2 are the centroids of the N1–N3/C3/C5 and N4/C6–C10 rings, respectively] and an N6—O7⋯C1(*x*, −1 + *y*, *z*) weak inter­molecular inter­action [with an O⋯C distance of 2.913 (5) Å] is present, forming layers parallel to the (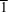
01) plane (Fig. 2 ▸).

The inter­molecular inter­actions in the crystal structure of the title compound were analysed using the *d*_norm_ property (Fig. S1) mapped over the Hirshfeld surface (Spackman & Jayatilaka, 2009 ▸), which was calculated using the *CrystalExplorer21* program (Spackman *et al.*, 2021 ▸). The strongest contacts, which are visualized on the Hirshfeld surface are the N—O⋯C inter­actions. The lighter red spots correspond to π-inter­actions. The majority of the inter­molecular inter­actions of the title compound are weak, and are represented in blue on the Hirshfeld surface.

For further exploration of the inter­molecular inter­actions, two-dimensional fingerprint plots (McKinnon *et al.*, 2007 ▸) were generated, as shown in Fig. S2. The major contributions to the crystal structure are from the H⋯H (39.9%) and H⋯O/O⋯H (37.9%) inter­actions. The H⋯C/C⋯H (6.9%), N⋯H/H⋯N (4.9%), N⋯C/C⋯N (3.5%), O⋯C/C⋯O (2.9%) and C⋯C (1.3%) inter­actions are less impactful in comparison.

## 4. Database survey

A search of the Cambridge Structural Database (CSD Version 5.46, updated November 2024; Groom *et al.*, 2016 ▸) yielded twelve structures of lanthanide complexes with coordination number 12 and coordinated by three NO_3_^−^ groups. Among them three structures with the La atom [refcodes AWAKER (Liu *et al.*, 2021 ▸), MILWEJ (Liu *et al.*, 2001 ▸), UBAMUI (Raja *et al.*, 2016*a* ▸)], six structures with the Ce atom [refcodes FOTXOC (Zhang & Liu, 2009 ▸), HIXWEQ (Christidis *et al.*, 1999 ▸), HOZQOF (Lin *et al.*, 2019 ▸), JORLIO (Nakase *et al.*, 2018 ▸), USEBAX (Zhao *et al.*, 2016 ▸), VAPDIC (Raja *et al.*, 2016*b* ▸)], and three structures with the Pr atom [refcodes KERPEF (Reddy *et al.*, 2017 ▸), PICSON (Gueye *et al.*, 2022 ▸), VIMWAR (Panayiotidou *et al.*, 2013 ▸)]. In the coordination polyhedrons of these structures, the Ln—O and Ln—N bond distances vary from 2.589–2.728 Å and 2.656–2.937 Å, respectively.

## 5. Synthesis and crystallization

Et-MPTA was synthesized according to a previously described procedure (Kharlova *et al.*, 2019 ▸; Khomenko *et al.*, 2016 ▸). For the synthesis of the La(Et-MPTA)_2_(NO_3_)_3_ complex, 0.2 mmol (0.0866 g) of La(NO_3_)_3_·6H_2_O and 0.4 mmol (0.0984 g) of the Et-MPTA ligand were dissolved separately in approximately 5 mL of methanol under heating. The methano­lic solutions of La(NO_3_)_3_·6H_2_O and the ligand were combined in a 25 mL beaker and heated for 1–2 h on a magnetic stirrer with constant non-turbulent stirring, avoiding boiling the reaction mixture. The solution was cooled to room temperature with the beaker kept open; the final volume was 6-7 mL. Over the next few hours, crystallization was observed. In order to study the structure, the crystals were used together with the mother liquor. For further analysis, the obtained crystals were separated from the solution, washed, and dried. The crystals are soluble in methanol, ethanol, and insoluble in water. IR (KBr), cm^−1^: 3370 *m*, *br* (ν_OH_ stretching, adsorbed H_2_O), 2963 *w* (ν_CH_ stretching, alk­yl), 1740 *s* (ν_C=O_ stretching), 1605 *m* (ν_C=C_, ν_C=N_, stretching, aromatic), 1490 *s* (ν_4_ stretching, NO_3_), 1384 *s* (δ_C-H_, scissoring CH_2_), 1324 *s* (ν_1_ stretching, NO_3_), 1034 *m* (ν_3_ stretching, NO_3_), 1194 *m* (ν_C–O_ stretching, ether), 1034 *w* (ν_C-N_ stretching, ring, δ_C–H_ bending), 566 *w* (ν_La–O_ stretching), 434 *w* (ν_La–N_ stretching).

## 6. Refinement

Crystal data, data collection and structure refinement details are summarized in Table 2 ▸. The H atoms were placed in calculated positions and refined using a riding model with *U*_iso_(H) = *nU*_eq_ of the carrier atom (*n* = 1.5 for methyl groups and *n* = 1.2 for other hydrogen atoms). The C atoms of the ethyl group are disordered over two positions with an occupancy of 50%. Restraints were applied to the bond lengths in the disordered parts (O—C = 1.420 Å, C—C = 1.513 Å) within a standard deviation of 0.05 Å. 

## Supplementary Material

Crystal structure: contains datablock(s) I. DOI: 10.1107/S2056989025005419/oo2010sup1.cif

Structure factors: contains datablock(s) I. DOI: 10.1107/S2056989025005419/oo2010Isup2.hkl

Supporting Information Figure S1. DOI: 10.1107/S2056989025005419/oo2010sup4.tif

Supporting information Figure S2. DOI: 10.1107/S2056989025005419/oo2010sup5.tif

Supporting information file containing figures for Hirshfeld surface analysis. DOI: 10.1107/S2056989025005419/oo2010sup6.doc

CCDC reference: 2465028

Additional supporting information:  crystallographic information; 3D view; checkCIF report

## Figures and Tables

**Table 1:** Selected bond lengths (Å)

La1—O1^i^	2.696 (2)	La1—O6^i^	2.648 (2)
La1—O1	2.696 (2)	La1—O6	2.648 (2)
La1—O3	2.658 (2)	La1—N1^i^	2.735 (2)
La1—O3^i^	2.658 (2)	La1—N1	2.735 (2)
La1—O4	2.752 (2)	La1—N4^i^	2.841 (2)
La1—O4^i^	2.752 (2)	La1—N4	2.841 (2)

Symmetry code: (i) 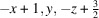.

**Table 2:** Experimental details

Crystal data	
Chemical formula	[La(NO_3_)_3_(C_12_H_14_N_4_O_2_)_2_]
*M*_r_	817.48
Crystal system, space group	Monoclinic, *C*2/*c*
Temperature (K)	296
*a*, *b*, *c* (Å)	20.9094 (15), 9.0361 (5), 19.3753 (13)
β (°)	122.360 (8)
*V* (Å^3^)	3092.2 (4)
*Z*	4
Radiation type	Mo *K*α
μ (mm^−1^)	1.47
Crystal size (mm)	0.20 × 0.1 × 0.08

Data collection	
Diffractometer	Bruker APEXII CCD
Absorption correction	Multi-scan (*SADABS*; Krause *et al.*, 2015 ▸)
*T*_min_, *T*_max_	0.631, 0.746
No. of measured, independent and observed [*I* > 2σ(*I*)] reflections	25399, 3551, 3079
*R*_int_	0.066
(sin θ/λ)_max_ (Å^−1^)	0.649

Refinement	
*R*[*F*^2^ > 2σ(*F*^2^)], *wR*(*F*^2^), *S*	0.035, 0.062, 1.03
No. of reflections	3551
No. of parameters	244
No. of restraints	4
H-atom treatment	H-atom parameters constrained
Δρ_max_, Δρ_min_ (e Å^−3^)	0.57, −0.84

Computer programs: *APEX2* and *SAINT* (Bruker, 2014 ▸), *SHELXT2018/2* (Sheldrick, 2015*a* ▸), *SHELXL2019/3* (Sheldrick, 2015*b* ▸) and *OLEX2* (Dolomanov *et al.*, 2009 ▸).

## supplementary crystallographic information

### \ Bis{ethyl 2-[1-methyl-3-(pyridin-2-yl)-1H-1,2,4-triazol-5-yl]acetate}\ trinitratolanthanum(III) Crystal data

**Table:**

[La(NO_3_)_3_(C_12_H_14_N_4_O_2_)_2_]	*F*(000) = 1640
*M**_r_* = 817.48	*D*_x_ = 1.756 Mg m^−^^3^
Monoclinic, *C*2/*c*	Mo *K*α radiation, λ = 0.71073 Å
*a* = 20.9094 (15) Å	Cell parameters from 6040 reflections
*b* = 9.0361 (5) Å	θ = 2.3–29.5°
*c* = 19.3753 (13) Å	µ = 1.47 mm^−^^1^
β = 122.360 (8)°	*T* = 296 K
*V* = 3092.2 (4) Å^3^	Block, colourless
*Z* = 4	0.20 × 0.1 × 0.08 mm

### \ Bis{ethyl 2-[1-methyl-3-(pyridin-2-yl)-1H-1,2,4-triazol-5-yl]acetate}\ trinitratolanthanum(III) Data collection

**Table:**

Bruker APEXII CCD diffractometer	3079 reflections with *I* > 2σ(*I*)
Graphite monochromator	*R*_int_ = 0.066
φ and ω scans	θ_max_ = 27.5°, θ_min_ = 2.3°
Absorption correction: multi-scan (SADABS; Krause *et al.*, 2015)	*h* = −27→27
*T*_min_ = 0.631, *T*_max_ = 0.746	*k* = −11→11
25399 measured reflections	*l* = −25→23
3551 independent reflections	

### \ Bis{ethyl 2-[1-methyl-3-(pyridin-2-yl)-1H-1,2,4-triazol-5-yl]acetate}\ trinitratolanthanum(III) Refinement

**Table:**

Refinement on *F*^2^	Primary atom site location: dual
Least-squares matrix: full	Hydrogen site location: inferred from neighbouring sites
*R*[*F*^2^ > 2σ(*F*^2^)] = 0.035	H-atom parameters constrained
*wR*(*F*^2^) = 0.062	*w* = 1/[σ^2^(*F*_o_^2^) + (0.0207*P*)^2^ + 5.1659*P*] where *P* = (*F*_o_^2^ + 2*F*_c_^2^)/3
*S* = 1.02	(Δ/σ)_max_ = 0.001
3551 reflections	Δρ_max_ = 0.57 e Å^−^^3^
244 parameters	Δρ_min_ = −0.84 e Å^−^^3^
4 restraints	

### \ Bis{ethyl 2-[1-methyl-3-(pyridin-2-yl)-1H-1,2,4-triazol-5-yl]acetate}\ trinitratolanthanum(III) Special details

**Table:**

Geometry. All esds (except the esd in the dihedral angle between two l.s. planes) are estimated using the full covariance matrix. The cell esds are taken into account individually in the estimation of esds in distances, angles and torsion angles; correlations between esds in cell parameters are only used when they are defined by crystal symmetry. An approximate (isotropic) treatment of cell esds is used for estimating esds involving l.s. planes.

### \ Bis{ethyl 2-[1-methyl-3-(pyridin-2-yl)-1H-1,2,4-triazol-5-yl]acetate}\ trinitratolanthanum(III) Fractional atomic coordinates and isotropic or equivalent isotropic displacement parameters (Å^2^)

**Table:**

	*x*	*y*	*z*	*U*_iso_*/*U*_eq_	Occ. (<1)
La1	0.500000	0.70697 (3)	0.750000	0.02422 (8)	
O1	0.49470 (11)	0.9461 (2)	0.83001 (13)	0.0342 (5)	
O2	0.47068 (12)	1.1356 (3)	0.88686 (13)	0.0490 (6)	
O3	0.41282 (12)	0.6737 (2)	0.81036 (13)	0.0420 (6)	
O4	0.36568 (13)	0.5606 (3)	0.69543 (14)	0.0450 (6)	
O5	0.30520 (15)	0.5608 (4)	0.75756 (18)	0.0757 (9)	
O6	0.48997 (14)	0.4392 (2)	0.68942 (13)	0.0446 (6)	
O7	0.500000	0.2329 (4)	0.750000	0.1035 (19)	
N1	0.37318 (13)	0.8792 (3)	0.66653 (14)	0.0302 (6)	
N2	0.30215 (14)	1.0757 (3)	0.62102 (17)	0.0396 (7)	
N3	0.29540 (14)	1.0126 (3)	0.55397 (16)	0.0385 (6)	
N4	0.40813 (13)	0.6946 (3)	0.57673 (14)	0.0306 (5)	
N5	0.35979 (15)	0.5972 (3)	0.75405 (17)	0.0415 (7)	
N6	0.500000	0.3676 (4)	0.750000	0.0504 (11)	
C1	0.45126 (17)	1.0320 (3)	0.83070 (18)	0.0335 (7)	
C2	0.36730 (18)	1.0331 (4)	0.7704 (2)	0.0453 (9)	
H2A	0.343480	0.962493	0.787535	0.054*	
H2B	0.347431	1.130488	0.769625	0.054*	
C3	0.34823 (16)	0.9948 (3)	0.68711 (19)	0.0330 (7)	
C4	0.2648 (2)	1.2166 (5)	0.6141 (3)	0.0697 (12)	
H4A	0.300776	1.295679	0.631030	0.105*	
H4B	0.244539	1.214648	0.648449	0.105*	
H4C	0.224384	1.231862	0.558420	0.105*	
C5	0.33897 (15)	0.8949 (3)	0.58451 (17)	0.0288 (6)	
C6	0.35208 (15)	0.7916 (3)	0.53514 (17)	0.0312 (6)	
C7	0.30868 (17)	0.7975 (4)	0.45033 (18)	0.0396 (7)	
H7	0.269901	0.866415	0.423460	0.048*	
C8	0.32411 (19)	0.6997 (4)	0.40704 (19)	0.0447 (8)	
H8	0.296155	0.701751	0.350232	0.054*	
C9	0.3815 (2)	0.5987 (4)	0.4488 (2)	0.0460 (9)	
H9	0.392798	0.530563	0.420863	0.055*	
C10	0.42195 (19)	0.6004 (4)	0.53292 (19)	0.0411 (8)	
H10	0.461029	0.532385	0.560819	0.049*	
C11A	0.5493 (3)	1.1248 (13)	0.9524 (5)	0.047 (3)	0.5
H11A	0.581712	1.165465	0.935363	0.057*	0.5
H11B	0.563293	1.022301	0.968023	0.057*	0.5
C11B	0.5489 (3)	1.1767 (13)	0.9403 (5)	0.053 (4)	0.5
H11C	0.554468	1.283395	0.940967	0.063*	0.5
H11D	0.579518	1.132313	0.921904	0.063*	0.5
C12A	0.5574 (5)	1.2123 (9)	1.0228 (5)	0.0440 (19)	0.5
H12A	0.541187	1.312379	1.005664	0.066*	0.5
H12B	0.609401	1.212011	1.067173	0.066*	0.5
H12C	0.526652	1.168387	1.040412	0.066*	0.5
C12B	0.5734 (5)	1.1211 (11)	1.0242 (6)	0.068 (3)	0.5
H12D	0.565756	1.016096	1.022133	0.102*	0.5
H12E	0.544112	1.168907	1.042468	0.102*	0.5
H12F	0.626122	1.142940	1.061360	0.102*	0.5

### \ Bis{ethyl 2-[1-methyl-3-(pyridin-2-yl)-1H-1,2,4-triazol-5-yl]acetate}\ trinitratolanthanum(III) Atomic displacement parameters (Å^2^)

**Table:**

	*U*^11^	*U*^22^	*U*^33^	*U*^12^	*U*^13^	*U*^23^
La1	0.02401 (12)	0.02188 (12)	0.02192 (12)	0.000	0.00906 (10)	0.000
O1	0.0289 (11)	0.0289 (11)	0.0396 (13)	0.0067 (9)	0.0150 (10)	0.0011 (9)
O2	0.0382 (13)	0.0619 (16)	0.0406 (14)	0.0085 (12)	0.0169 (12)	−0.0151 (12)
O3	0.0405 (13)	0.0439 (14)	0.0374 (13)	−0.0049 (10)	0.0180 (11)	−0.0037 (10)
O4	0.0427 (14)	0.0476 (15)	0.0392 (14)	−0.0067 (11)	0.0184 (12)	−0.0029 (11)
O5	0.0450 (16)	0.116 (3)	0.072 (2)	−0.0192 (16)	0.0356 (15)	0.0128 (18)
O6	0.0654 (16)	0.0292 (12)	0.0372 (13)	−0.0023 (11)	0.0261 (12)	0.0024 (10)
O7	0.228 (6)	0.024 (2)	0.098 (4)	0.000	0.113 (4)	0.000
N1	0.0252 (12)	0.0337 (14)	0.0289 (14)	0.0029 (11)	0.0126 (11)	0.0011 (11)
N2	0.0308 (14)	0.0368 (15)	0.0445 (17)	0.0100 (12)	0.0157 (13)	0.0054 (13)
N3	0.0347 (15)	0.0401 (16)	0.0342 (15)	0.0074 (12)	0.0141 (13)	0.0072 (12)
N4	0.0318 (13)	0.0277 (13)	0.0273 (12)	−0.0014 (11)	0.0126 (11)	0.0007 (11)
N5	0.0320 (15)	0.0463 (17)	0.0420 (17)	−0.0012 (13)	0.0170 (14)	0.0116 (14)
N6	0.081 (3)	0.022 (2)	0.057 (3)	0.000	0.042 (3)	0.000
C1	0.0336 (17)	0.0358 (17)	0.0283 (16)	0.0057 (14)	0.0146 (14)	0.0017 (14)
C2	0.0339 (18)	0.057 (2)	0.040 (2)	0.0081 (17)	0.0167 (16)	−0.0074 (17)
C3	0.0202 (15)	0.0351 (17)	0.0351 (18)	0.0018 (13)	0.0091 (14)	0.0015 (14)
C4	0.076 (3)	0.058 (3)	0.069 (3)	0.041 (2)	0.035 (2)	0.014 (2)
C5	0.0207 (14)	0.0300 (15)	0.0293 (16)	−0.0024 (12)	0.0092 (13)	0.0045 (13)
C6	0.0248 (14)	0.0337 (15)	0.0291 (15)	−0.0082 (14)	0.0104 (12)	0.0019 (15)
C7	0.0315 (16)	0.0482 (19)	0.0288 (16)	−0.0036 (16)	0.0092 (14)	0.0068 (16)
C8	0.0438 (19)	0.057 (2)	0.0254 (16)	−0.0148 (19)	0.0135 (15)	−0.0045 (17)
C9	0.054 (2)	0.049 (2)	0.0366 (19)	−0.0063 (18)	0.0252 (18)	−0.0093 (17)
C10	0.046 (2)	0.0388 (18)	0.0320 (18)	0.0020 (16)	0.0165 (16)	−0.0023 (15)
C11A	0.029 (5)	0.070 (9)	0.030 (5)	0.006 (4)	0.008 (4)	−0.021 (5)
C11B	0.049 (6)	0.045 (7)	0.058 (7)	−0.010 (4)	0.025 (5)	−0.011 (5)
C12A	0.050 (5)	0.045 (4)	0.038 (4)	−0.003 (4)	0.024 (4)	−0.014 (4)
C12B	0.057 (6)	0.082 (7)	0.053 (6)	−0.027 (6)	0.022 (5)	0.001 (6)

### \ Bis{ethyl 2-[1-methyl-3-(pyridin-2-yl)-1H-1,2,4-triazol-5-yl]acetate}\ trinitratolanthanum(III) Geometric parameters (Å, º)

**Table:**

La1—O1^i^	2.696 (2)	C1—C2	1.500 (4)
La1—O1	2.696 (2)	C2—H2A	0.9700
La1—O3	2.658 (2)	C2—H2B	0.9700
La1—O3^i^	2.658 (2)	C2—C3	1.482 (4)
La1—O4	2.752 (2)	C4—H4A	0.9600
La1—O4^i^	2.752 (2)	C4—H4B	0.9600
La1—O6^i^	2.648 (2)	C4—H4C	0.9600
La1—O6	2.648 (2)	C5—C6	1.464 (4)
La1—N1^i^	2.735 (2)	C6—C7	1.389 (4)
La1—N1	2.735 (2)	C7—H7	0.9300
La1—N4^i^	2.841 (2)	C7—C8	1.370 (5)
La1—N4	2.841 (2)	C8—H8	0.9300
O1—C1	1.200 (3)	C8—C9	1.375 (5)
O2—C1	1.323 (4)	C9—H9	0.9300
O2—C11A	1.446 (5)	C9—C10	1.377 (4)
O2—C11B	1.440 (5)	C10—H10	0.9300
O3—N5	1.266 (3)	C11A—H11A	0.9700
O4—N5	1.251 (3)	C11A—H11B	0.9700
O5—N5	1.224 (3)	C11A—C12A	1.506 (5)
O6—N6	1.256 (3)	C11B—H11C	0.9700
O7—N6	1.217 (5)	C11B—H11D	0.9700
N1—C3	1.321 (4)	C11B—C12B	1.505 (5)
N1—C5	1.357 (3)	C12A—H12A	0.9600
N2—N3	1.355 (4)	C12A—H12B	0.9600
N2—C3	1.335 (4)	C12A—H12C	0.9600
N2—C4	1.462 (4)	C12B—H12D	0.9600
N3—C5	1.317 (4)	C12B—H12E	0.9600
N4—C6	1.334 (4)	C12B—H12F	0.9600
N4—C10	1.339 (4)		
			
O1—La1—O1^i^	73.45 (9)	C3—N2—N3	109.9 (3)
O1^i^—La1—O4	120.78 (6)	C3—N2—C4	129.8 (3)
O1^i^—La1—O4^i^	104.97 (7)	C5—N3—N2	102.5 (2)
O1—La1—O4	104.97 (7)	C6—N4—La1	120.53 (19)
O1—La1—O4^i^	120.78 (6)	C6—N4—C10	116.9 (3)
O1^i^—La1—N1	61.91 (7)	C10—N4—La1	122.4 (2)
O1^i^—La1—N1^i^	63.81 (7)	O4—N5—O3	117.4 (3)
O1—La1—N1^i^	61.91 (7)	O5—N5—O3	120.8 (3)
O1—La1—N1	63.81 (7)	O5—N5—O4	121.8 (3)
O1^i^—La1—N4^i^	120.07 (7)	O6—N6—La1	58.98 (18)
O1—La1—N4	120.07 (7)	O6^i^—N6—La1	58.98 (18)
O1^i^—La1—N4	64.01 (7)	O6^i^—N6—O6	118.0 (4)
O1—La1—N4^i^	64.01 (7)	O7—N6—La1	180.0
O3—La1—O1	65.72 (6)	O7—N6—O6	121.02 (18)
O3^i^—La1—O1^i^	65.72 (6)	O7—N6—O6^i^	121.02 (18)
O3^i^—La1—O1	126.35 (6)	O1—C1—O2	124.4 (3)
O3—La1—O1^i^	126.35 (6)	O1—C1—C2	124.9 (3)
O3—La1—O3^i^	167.00 (9)	O2—C1—C2	110.6 (3)
O3^i^—La1—O4	125.16 (7)	C1—C2—H2A	109.3
O3^i^—La1—O4^i^	46.80 (7)	C1—C2—H2B	109.3
O3—La1—O4	46.80 (7)	H2A—C2—H2B	108.0
O3—La1—O4^i^	125.16 (7)	C3—C2—C1	111.5 (3)
O3—La1—N1^i^	118.72 (7)	C3—C2—H2A	109.3
O3^i^—La1—N1^i^	69.40 (7)	C3—C2—H2B	109.3
O3^i^—La1—N1	118.72 (7)	N1—C3—N2	110.0 (3)
O3—La1—N1	69.40 (7)	N1—C3—C2	126.4 (3)
O3—La1—N4^i^	70.42 (7)	N2—C3—C2	123.5 (3)
O3—La1—N4	109.05 (7)	N2—C4—H4A	109.5
O3^i^—La1—N4^i^	109.05 (7)	N2—C4—H4B	109.5
O3^i^—La1—N4	70.41 (7)	N2—C4—H4C	109.5
O4—La1—O4^i^	122.58 (10)	H4A—C4—H4B	109.5
O4—La1—N4	67.77 (7)	H4A—C4—H4C	109.5
O4^i^—La1—N4^i^	67.76 (7)	H4B—C4—H4C	109.5
O4—La1—N4^i^	109.92 (7)	N1—C5—C6	122.2 (3)
O4^i^—La1—N4	109.92 (7)	N3—C5—N1	114.5 (3)
O6—La1—O1^i^	119.69 (7)	N3—C5—C6	123.2 (3)
O6^i^—La1—O1^i^	165.76 (6)	N4—C6—C5	115.8 (2)
O6—La1—O1	165.77 (6)	N4—C6—C7	123.1 (3)
O6^i^—La1—O1	119.70 (7)	C7—C6—C5	121.1 (3)
O6—La1—O3	100.45 (7)	C6—C7—H7	120.6
O6^i^—La1—O3^i^	100.45 (7)	C8—C7—C6	118.7 (3)
O6^i^—La1—O3	67.16 (7)	C8—C7—H7	120.6
O6—La1—O3^i^	67.16 (7)	C7—C8—H8	120.5
O6—La1—O4	64.43 (7)	C7—C8—C9	119.0 (3)
O6—La1—O4^i^	63.50 (7)	C9—C8—H8	120.5
O6^i^—La1—O4	63.50 (7)	C8—C9—H9	120.7
O6^i^—La1—O4^i^	64.43 (7)	C8—C9—C10	118.6 (3)
O6^i^—La1—O6	47.95 (9)	C10—C9—H9	120.7
O6^i^—La1—N1	127.14 (7)	N4—C10—C9	123.6 (3)
O6—La1—N1	115.86 (7)	N4—C10—H10	118.2
O6^i^—La1—N1^i^	115.86 (7)	C9—C10—H10	118.2
O6—La1—N1^i^	127.14 (7)	O2—C11A—H11A	110.4
O6^i^—La1—N4	109.33 (7)	O2—C11A—H11B	110.4
O6^i^—La1—N4^i^	66.26 (7)	O2—C11A—C12A	106.5 (6)
O6—La1—N4	66.25 (7)	H11A—C11A—H11B	108.6
O6—La1—N4^i^	109.33 (7)	C12A—C11A—H11A	110.4
N1—La1—O4^i^	165.39 (7)	C12A—C11A—H11B	110.4
N1^i^—La1—O4^i^	65.10 (7)	O2—C11B—H11C	110.4
N1—La1—O4	65.11 (7)	O2—C11B—H11D	110.4
N1^i^—La1—O4	165.39 (7)	O2—C11B—C12B	106.8 (7)
N1—La1—N1^i^	110.63 (10)	H11C—C11B—H11D	108.6
N1^i^—La1—N4	123.34 (7)	C12B—C11B—H11C	110.4
N1—La1—N4^i^	123.34 (7)	C12B—C11B—H11D	110.4
N1—La1—N4	59.67 (7)	C11A—C12A—H12A	109.5
N1^i^—La1—N4^i^	59.67 (7)	C11A—C12A—H12B	109.5
N4—La1—N4^i^	175.51 (10)	C11A—C12A—H12C	109.5
C1—O1—La1	142.1 (2)	H12A—C12A—H12B	109.5
C1—O2—C11A	112.3 (4)	H12A—C12A—H12C	109.5
C1—O2—C11B	120.3 (6)	H12B—C12A—H12C	109.5
N5—O3—La1	99.98 (17)	C11B—C12B—H12D	109.5
N5—O4—La1	95.78 (17)	C11B—C12B—H12E	109.5
N6—O6—La1	97.0 (2)	C11B—C12B—H12F	109.5
C3—N1—La1	133.32 (19)	H12D—C12B—H12E	109.5
C3—N1—C5	103.0 (2)	H12D—C12B—H12F	109.5
C5—N1—La1	119.27 (18)	H12E—C12B—H12F	109.5
N3—N2—C4	120.2 (3)		
			
La1—O1—C1—O2	174.4 (2)	N4—C6—C7—C8	−0.3 (5)
La1—O1—C1—C2	−2.8 (5)	C1—O2—C11A—C12A	−162.2 (7)
La1—O3—N5—O4	2.3 (3)	C1—O2—C11B—C12B	−110.0 (8)
La1—O3—N5—O5	−177.9 (3)	C1—C2—C3—N1	50.7 (4)
La1—O4—N5—O3	−2.2 (3)	C1—C2—C3—N2	−129.2 (3)
La1—O4—N5—O5	178.0 (3)	C3—N1—C5—N3	0.4 (3)
La1—O6—N6—O6^i^	−0.003 (3)	C3—N1—C5—C6	178.3 (3)
La1—O6—N6—O7	180.000 (2)	C3—N2—N3—C5	−0.1 (3)
La1—N1—C3—N2	154.6 (2)	C4—N2—N3—C5	176.8 (3)
La1—N1—C3—C2	−25.3 (5)	C4—N2—C3—N1	−176.1 (3)
La1—N1—C5—N3	−159.02 (19)	C4—N2—C3—C2	3.8 (5)
La1—N1—C5—C6	18.9 (3)	C5—N1—C3—N2	−0.5 (3)
La1—N4—C6—C5	−3.8 (3)	C5—N1—C3—C2	179.6 (3)
La1—N4—C6—C7	175.5 (2)	C5—C6—C7—C8	178.9 (3)
La1—N4—C10—C9	−175.5 (3)	C6—N4—C10—C9	−0.4 (5)
O1—C1—C2—C3	−37.0 (5)	C6—C7—C8—C9	0.5 (5)
O2—C1—C2—C3	145.4 (3)	C7—C8—C9—C10	−0.6 (5)
N1—C5—C6—N4	−9.9 (4)	C8—C9—C10—N4	0.6 (5)
N1—C5—C6—C7	170.8 (3)	C10—N4—C6—C5	−179.0 (3)
N2—N3—C5—N1	−0.2 (3)	C10—N4—C6—C7	0.2 (4)
N2—N3—C5—C6	−178.1 (3)	C11A—O2—C1—O1	−5.7 (7)
N3—N2—C3—N1	0.4 (3)	C11A—O2—C1—C2	171.9 (6)
N3—N2—C3—C2	−179.7 (3)	C11B—O2—C1—O1	15.8 (6)
N3—C5—C6—N4	167.8 (3)	C11B—O2—C1—C2	−166.6 (5)
N3—C5—C6—C7	−11.4 (4)		

Symmetry code: (i) −*x*+1, *y*, −*z*+3/2.
