# Supplementary figures and images for: Crystal structure and supra­molecular features of bis­{ethyl 2-[1-methyl-3-(pyridin-2-yl)-1H-1,2,4-triazol-5-yl]acetate}­tri­nitratolanthanum(III)
Source: Acta Crystallogr E Crystallogr Commun. 2025 Jun 24;81(Pt 7):632–5. doi: 10.1107/S2056989025005419 (PMC12230605; doi:10.1107/S2056989025005419)

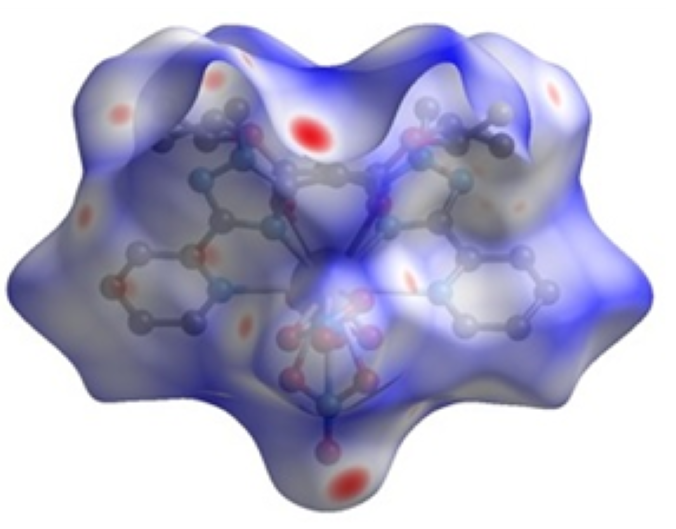

Supplement: Supplementary file 3 [file e-81-00632-sup4.tif]

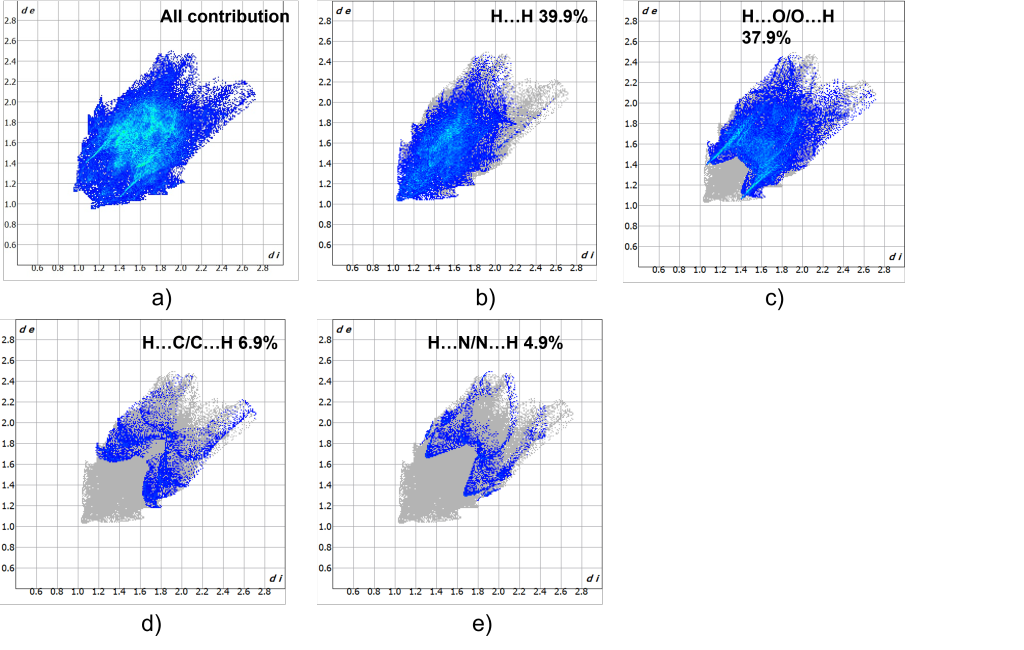

Supplement: Supplementary file 4 [file e-81-00632-sup5.tif]
