# Supplementary material for: Crystal structure and supra­molecular features of bis­{ethyl 2-[1-methyl-3-(pyridin-2-yl)-1H-1,2,4-triazol-5-yl]acetate}­tri­nitratolanthanum(III)
Source: Acta Crystallogr E Crystallogr Commun. 2025 Jun 24;81(Pt 7):632–5. doi: 10.1107/S2056989025005419 (PMC12230605; doi:10.1107/S2056989025005419)
Supplement: Supplementary file 5 [file e-81-00632-sup6.doc]

**Supporting information**


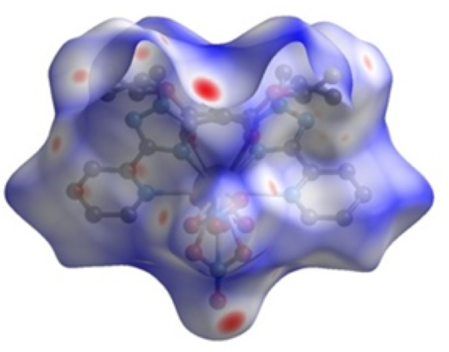


[Figure S1](../../readonly). The Hirshfeld surface mapped overdnormfor visualizing the intermolecular contacts of compound La(Et-MPTA)_2_(NO_3_)_3_


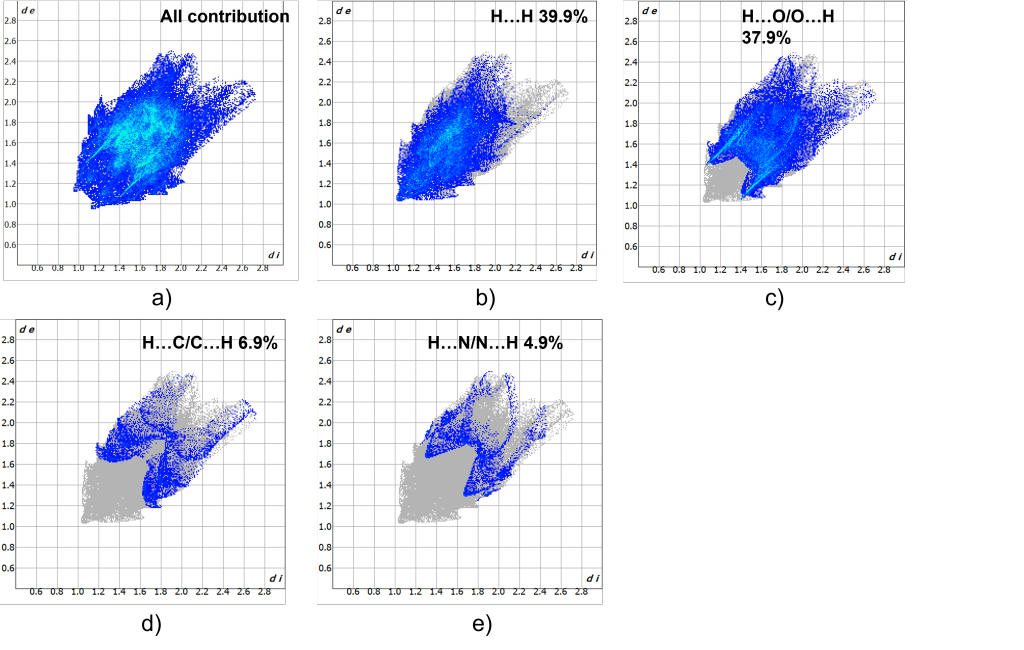


[Figure S2](../../readonly). Two-dimensional fingerprint plots for La(Et-MPTA)_2_(NO_3_)_3_ showing (*a*) all interactions, and (*b*)–(*e*) delineated into contributions from other contacts (blue areas) [*d*_e_ and *d*_i_ represent the distances from a point on the Hirshfeld surface to the nearest atoms outside (external) and inside (internal) the surface, respectively].
